# Progress of nursing management for delirium in critically ill geriatric patients: a narrative review

**DOI:** 10.3389/fnhum.2026.1849325

**Published:** 2026-09-22

**Authors:** Qi Mei, Xinyi Chen

**Affiliations:** 1 Intensive Care Unit, Shanghai Eighth People’s Hospital, Shanghai, China; 2 Outpatient Office, Shanghai Eighth People’s Hospital, Shanghai, China

**Keywords:** cognitive impairment, critically ill patients, elderly, multicomponent nonpharmacological intervention, nursing

## Abstract

Delirium is the most common and modifiable acute cognitive complication in elderly critically ill patients and is associated with prolonged hospital stays, increased mortality, persistent long-term cognitive decline, and a significant healthcare burden. A history of mild cognitive impairment or dementia further increases delirium risk, exacerbates symptom severity, and worsens clinical outcomes. Nonpharmacological nursing interventions form the cornerstone of delirium prevention and management. However, nurse-centered integrated comprehensive care pathways remain insufficiently integrated. This narrative review systematically synthesizes peer-reviewed evidence retrieved from the PubMed, Web of Science, and Scopus databases up to July 2025, focusing on the nursing management of delirium in elderly critically ill patients. The content encompasses modifiable risk factors, validated bedside assessment scales (including CAM-ICU, ICDSC, 4AT, and home-based tools such as FAM-CAM and IQCODE), and evidence-based multicomponent nonpharmacological intervention strategies. The core intervention domains include the Hospital Elder Life Program (HELP), the eCASH care model, medication safety monitoring, early mobilization, sleep optimization, reduction in physical restraints, orientation training, behavioral symptom management, and postdischarge home safety guidance. Additionally, this review evaluates the current status, clinical value, and limitations of emerging digital technologies applied to delirium care, including wearable monitoring devices, virtual reality-assisted cognitive rehabilitation, and artificial intelligence-augmented clinical decision support systems. This review aims to provide a theoretical foundation and practical reference for frontline nurses implementing evidence-based delirium management in elderly critically ill patients.

## 1 Introduction

Cognitive impairment is a dysfunction of the cerebral cortex that affects core cognitive domains, including memory, language, logical reasoning, orientation, and visual information processing, and is often accompanied by behavioral and psychological abnormalities (Wilcox et al., 2013). Elderly critically ill patients are particularly at high risk, with their cognitive decline stemming from the direct neurobiological impact of acute illness and physiological stress caused by intensive medical interventions (Pandharipande et al., 2014; Rengel et al., 2019). In the absence of timely intervention, it severely impairs patients’ social functioning and overall recovery, posing a significant public health challenge in the context of global population aging.

Cognitive impairment in elderly individuals encompasses three primary conditions: mild cognitive impairment (MCI), delirium (Schieveld et al., 2019), and dementia (Sachdev et al., 2014). The global prevalence of MCI in the elderly population is 23.7% (Salari et al., 2025), with its incidence increasing with age, reaching 6.7% in individuals aged 60–64 years and 25.2% in those aged 80–84 years (Petersen et al., 2018). The overall comorbidity rate of dementia among Chinese elderly individuals has increased by 4.76% since 1990 (from 2.43%) (Liang et al., 2026). In intensive care settings, delirium represents the most common, acute, and modifiable form of cognitive dysfunction. In intensive care units (ICUs), the overall prevalence of delirium among ICU adults is 31.8%, and it can reach 50–80% in elderly critically ill, mechanically ventilated individuals aged ≥65 years (Krewulak et al., 2018). Notably, preexisting MCI and dementia are the strongest risk factors for delirium development in elderly critically ill patients and exacerbate delirium severity and worsen prognosis. Delirium in elderly critically ill patients is associated with increased complications, prolonged hospital stays, elevated mortality, placement in acute postcare facilities, and persistent long-term cognitive decline, thereby leading to significant healthcare expenditures and caregiver burden(Salluh et al., 2015; Fong and Inouye, 2022).

Although numerous reviews have explored the pathophysiology and medical management of delirium in the ICU, several important gaps remain. First, few reviews have systematically integrated a comprehensive care pathway from bedside risk stratification and assessment to in-hospital multicomponent interventions and postdischarge home safety guidance from a nurse-led perspective. Second, the clinical value of family-participatory assessment tools and emerging digital technologies has not been sufficiently integrated into nursing-focused reviews. Third, practical nursing adaptations for patients with a history of dementia are often overlooked in existing reviews. This narrative review synthesizes existing evidence on the nursing management of delirium in elderly critically ill patients. It systematically covers modifiable risk factors, validated assessment scales, multicomponent nonpharmacological intervention strategies, and emerging technologies, including wearable devices, virtual reality (VR), and artificial intelligence (AI)-assisted decision support. By integrating existing evidence and highlighting clinical relevance, this review aims to provide a theoretical foundation and practical guidance for frontline clinical practice.

## 2 Methods

This article is a narrative review based on published peer-reviewed literature. Through comprehensive literature retrieval, studies related to delirium, cognitive impairment, intensive care, elderly patients, and nursing interventions were identified.

### 2.1 Literature retrieval

Literature retrieval was conducted in English databases, including PubMed, Web of Science, and Scopus. The search scope covered literature published from the inception of each database up to July 2025.

### 2.2 Search terms

The following search terms were used to identify the literature: (“delirium” OR “cognitive impairment” OR “acute brain dysfunction”) AND (“critically ill” OR “intensive care unit” OR “ICU”) AND (“elderly” OR “geriatric” OR “older adults”) AND (“nursing” OR “nursing intervention” OR “nonpharmacological management”).

### 2.3 Inclusion and exclusion criteria

The inclusion criteria were as follows: (1) study population consisting of adult patients aged ≥60 years in an intensive care setting; (2) reported risk factors for delirium, assessment tools, or nursing interventions; (3) original research, systematic reviews, meta-analyses, or clinical practice guidelines; and (4) published in English.

The exclusion criteria were case reports, conference abstracts, letters, or non-English publications. Two authors independently screened titles and abstracts for eligibility, followed by full-text review of potentially relevant articles. Any discrepancies were resolved through discussion.

### 2.4 Study selection

The initial database search yielded 2,691 records. After removing 962 duplicates using EndNote, 1,729 unique records remained. Two independent reviewers screened these by title and abstract, excluding 1,431 records that focused on ineligible populations (e.g., pediatric or non-critically ill patients), did not address delirium care management, or fell outside the predefined study types (e.g., case reports, conference abstracts).

Subsequently, 298 full-text articles were retrieved for detailed full-text assessment. Of these, 201 articles were further excluded: 89 were case reports, letters or conference abstracts; 62 focused on patient populations outside the inclusion criteria; 31 lacked relevant nursing-oriented outcome data; and 19 were published in non-English languages. Disagreements among reviewers during the screening process were resolved through discussion; if consensus could not be reached, a third senior reviewer was consulted.

Ultimately, 97 publications were included for narrative synthesis, comprising 6 clinical practice guidelines, 35 systematic reviews and meta-analyses, 22 randomized controlled trials, 22 high-quality observational cohort studies, and 12 methodological studies. During evidence synthesis, high-quality systematic reviews, multicenter randomized controlled trials, and international clinical practice guidelines were prioritized to ensure the reliability and clinical applicability of the narrative conclusions.

## 3 Risk factors for delirium in elderly critically ill patients

Delirium in elderly critically ill patients is associated with substantial healthcare costs and increased caregiver burden. Early identification of modifiable risk factors enables nurses to implement targeted preventive interventions at the bedside, potentially reducing the incidence of delirium, shortening hospital stays, and lowering mortality rates (Szewczak et al., 2025). From a nursing perspective, recognizing predisposing and precipitating factors is the first step in delirium prevention. The main risk factors for delirium in elderly critically ill patients are summarized in Table 1 and illustrated in Figure 1.

**Table 1:** Main risk factors for delirium in elderly critically ill patients.

Category	Specific factors
Demographic factors	Age, living alone
Basic medical history	Diabetes mellitus, history of stroke
Medication factors	Benzodiazepines, opioid analgesics, anticholinergic agents, and systemic glucocorticoids
Lifestyles	Smoking and alcohol consumption
Inflammation biomarkers	CRP, IL-6, TNF-α, MMP-9, and NLR

**Figure 1:**
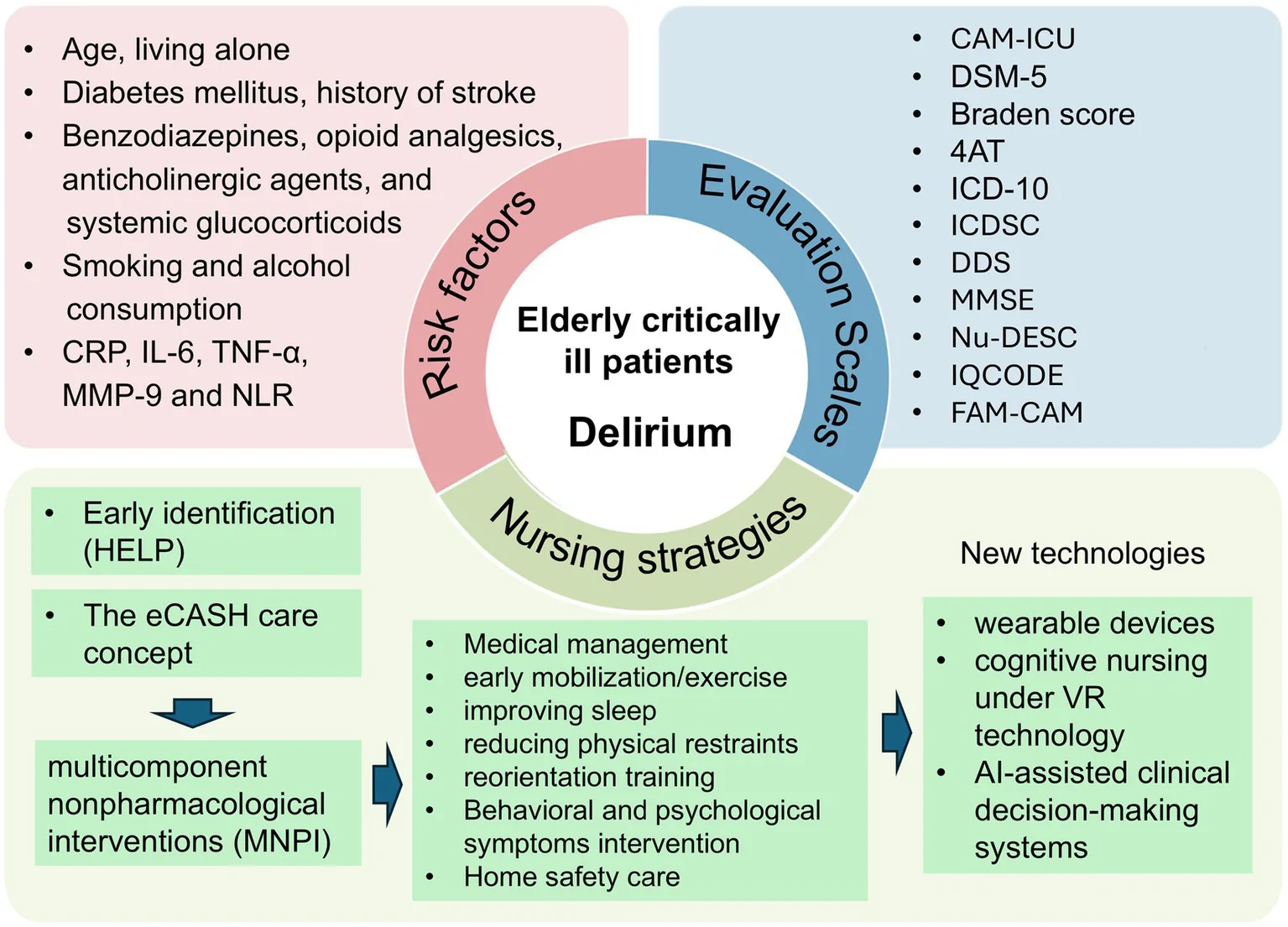
Risk factors, evaluation scales, and nursing strategies in elderly critically ill patients.

### 3.1 Age and living alone

Advanced age is one of the risk factors for delirium in elderly critically ill patients. Its incidence increases markedly in patients aged 65 years and older and increases further with advancing age (Schindele et al., 2026; Yates et al., 2025; Mailhot et al., 2022). Age-related cerebral vulnerability stems from multiple interrelated mechanisms: progressive neuronal loss, reduced synaptic plasticity, alterations in brain microstructure, impaired homeostatic regulation of cholinergic, dopaminergic and GABAergic neurotransmitter systems, and glial cell dysfunction (Fan et al., 2024; Bugiani, 2021; Pan and Wang, 2026; Heffernan et al., 2025). All these mechanisms diminish the capacity of the brain to withstand acute physiological stressors, such as hypoxia, hypotension and systemic inflammation, during critical illness.

Moreover, living alone and associated social isolation are significant social risk factors for delirium among elderly ICU patients admitted to the community (Vasunilashorn et al., 2025). Elderly individuals living alone often have undiagnosed underlying cognitive impairment or sensory deficits upon hospital admission and lack routine cognitive stimulation and emotional support from family members. Their risk of delirium is further elevated when they are exposed to a unique ICU environment characterized by circadian rhythm disruption, reduced social interaction, sensory overload or deprivation, and repeated invasive procedures (Rahimibashar et al., 2022; Pun et al., 2021). Against this backdrop, family voice intervention, as an adjuvant measure, has been proven to help prevent and reduce delirium in critically ill patients and may facilitate patient recovery as well as improve the mental health of family members (Peschel et al., 2026; Lee and Kang, 2025).

### 3.2 Diabetes mellitus and history of stroke

Among T2DM patients, there is a strong association between delirium and an increased risk of dementia in elderly individuals (≥65 years) (Sun et al., 2025). In patients with long-term diabetes, chronic hyperglycemia and insulin resistance impair neuronal function and synaptic plasticity, whereas microvascular complications lead to cerebral hypoperfusion and white matter lesions, compromising cognitive health (Chen et al., 2025). During critical illness, additional glucose fluctuations, insulin resistance, and oxidative stress further disrupt blood–brain barrier integrity and neurotransmitter balance, precipitating acute cognitive dysfunction (van Keulen et al., 2018).

Delirium is a common and serious complication of stroke during the acute stage in elderly patients (Shaw et al., 2019). Delirium adversely affects the prognosis of individuals with acute stroke. Those who experience poststroke delirium tend to have poorer functional outcomes and shorter survival times (Rollo et al., 2022). In a cohort of 698 patients (median age 73 years) with ischemic stroke or transient ischemic attack, compared with patients with inhibited delirium, those with advanced delirium were more likely to be preceded by irritability or emotional lability. All subtypes of delirium were associated with an increased risk of poorer functional outcomes at 3 and 12 months post-stroke, as well as an increased risk of mortality, compared with patients without delirium (Czyzycki et al., 2022). A history of ischemic or hemorrhagic stroke significantly increases the risk of delirium through permanent structural damage to cortical and subcortical neural networks, impaired cerebral autoregulation, and cognitive decline following prior strokes (Boukrina et al., 2022; Hao et al., 2025; Siokas et al., 2022).

### 3.3 Smoking and alcohol consumption

Smoking and alcohol consumption are important modifiable risk factors for delirium among older adults (Akbari, 2025). Chronic nicotine exposure impairs older adults’ ability to perform complex cognitive tasks, accelerates cognitive decline, and further increases the risk of delirium (Komninou et al., 2024). As gray matter regions are central to cognitive function, structural magnetic resonance imaging studies have consistently revealed significant gray matter atrophy among smokers. These neuroanatomical findings support the mechanistic pathway whereby smoking diminishes the cognitive reserve and predisposes individuals to delirium (Meysami et al., 2025).

Cognitive decline shows a dose-dependent relationship with alcohol intake (Topiwala and Ebmeier, 2018). Heavy alcohol use impairs brain health via multiple pathways: it damages neurons and synaptic connections, reduces the activity of key metabolic enzymes, and inhibits the absorption of thiamine (vitamin B1), collectively driving progressive cognitive dysfunction. Excessive alcohol consumption is associated with reductions in both gray matter and white matter volumes (Daviet et al., 2022). Collectively, these pathological alterations trigger progressive cognitive deterioration, rendering older individuals more vulnerable to acute delirium when confronted with physiological stress during critical illness (Park and Lee, 2019).

### 3.4 Surgery and medication-related factors

Surgery is an independent risk factor for delirium among older adults. Postoperative delirium (POD) develops in patients who have undergone surgical procedures and anesthesia and disproportionately affects elderly patients and those with preexisting cognitive impairment (Yan et al., 2023). POD is independently associated with an increased risk of mortality, postoperative complications, unplanned ICU admissions, prolonged hospital stay, and nonhome discharge in critically ill surgical patients (Yan et al., 2023; To-Adithep et al., 2023).

Medications are common and modifiable triggers for delirium, manifesting in three forms: the specific effects of a single agent, drug–drug interactions from polypharmacy, and adverse drug reactions. Alagiakrishnan et al. reported that approximately 12–39% of delirium cases in elderly patients are related to medical interventions (Alagiakrishnan and Wiens, 2004). Benzodiazepines are a particularly well-documented example: they are frequently administered perioperatively for anxiolysis and sleep and intraoperatively as premedication or anesthetic adjuvants (Proulx-Tremblay et al., 2020). While benzodiazepines may reduce intraoperative awareness because of their amnestic properties, a meta-analysis by Wang et al. revealed that perioperative benzodiazepine administration did not reduce intraoperative awareness and was associated with increased POD risk (Wang et al., 2023). Notably, benzodiazepine use in geriatric patients following major surgery is consistently linked to a higher incidence of POD (Duprey et al., 2022).

### 3.5 Inflammatory biomarkers

Peripheral inflammatory biomarkers function as objective biological predictors of delirium in critically ill elderly patients, offering empirical support for the neuroinflammatory hypothesis of acute cognitive dysfunction (Moorey et al., 2025; Noah et al., 2021). During critical illness, systemic inflammatory responses lead to elevated circulating levels of proinflammatory cytokines. These cytokines can cross the blood–brain barrier or activate circumventricular organs, thereby inducing microglial activation, oxidative stress, and widespread dysregulation of acetylcholine, dopamine, and GABA neurotransmitters (Liu and Zhang, 2025; Fan et al., 2024). Meta-analytic evidence from elderly ICU cohorts confirms that elevated baseline levels of C-reactive protein (CRP), interleukin-6 (IL-6), tumor necrosis factor-α (TNF-α), matrix metalloproteinase-9 (MMP-9), and the neutrophil-to-lymphocyte ratio (NLR) are significantly associated with an increased risk of incident delirium (Khan et al., 2023; Noah et al., 2021; Sarejloo et al., 2023). Furthermore, trajectories of persistent or increasing inflammatory markers during the ICU course are linked to longer delirium duration, more severe symptoms, and poorer long-term cognitive outcomes. Given that the elderly population exhibits age-related chronic low-grade inflammation, the inflammatory burden of critical illness further increases the risk of delirium (Forget et al., 2021).

## 4 Evaluation of cognitive impairments in critically ill geriatric patients

Accurate and timely assessments of delirium serve as the foundation for targeted nursing interventions (Figure 1). Given the fluctuating nature of delirium symptoms and the complexity of intensive care environments, standardized and validated scales are the primary methods for nurses to identify delirium at the bedside. The *Diagnostic and Statistical Manual of Mental Disorders, Fifth Edition* (DSM-5) (Sachdev et al., 2014) remains the reference standard for delirium diagnosis; however, owing to its complexity, it is not suitable for routine and frequent nursing assessments. Various validated screening tools have been developed for different clinical settings and patient populations.

The Confusion Assessment Method for the ICU (CAM-ICU), developed on the basis of the DSM-5, is the most widely used and validated delirium assessment tool in critical care settings. It has been translated into at least 12 languages and has been cited in more than 4,000 published studies. Research by Ely et al. confirmed the high reliability and accuracy of CAM-ICUs in mechanically ventilated patients (Ely et al., 2001). Inouye et al. reported that, with standard application, its sensitivity and specificity can reach 95% (Inouye et al., 1990). The Chinese version has also been demonstrated to possess good psychometric properties in clinical applications in China (Lin et al., 2004). However, the CAM-ICU has notable limitations. It requires standardized training for administrators to ensure consistent results, and its sensitivity to hypoactive delirium may decrease when it is used by less experienced staff (Dos Santos et al., 2022). It also relies on a certain level of patient cooperation to complete attention and thinking tasks. Therefore, the CAM-ICU is recommended as the first-line assessment tool for intubated and mechanically ventilated ICU patients, but it is not universally optimal across all clinical contexts(Miranda et al., 2023).

Several alternative tools are available for different clinical scenarios. The Intensive Care Delirium Screening Checklist (ICDSC) (Sakuramoto et al., 2015) is designed for ongoing screening during nursing shifts and has simple operation requirements but low overall diagnostic accuracy(Brummel et al., 2013). A meta-analysis by Gusmao-Flores et al. revealed that compared with CAM-ICUs, ICDSCs had lower sensitivity (74% vs. 80.0%), specificity (81.9% vs. 95.9%), and diagnostic odds ratios (21.5 vs. 103.2) in ICU patients (Gusmao-Flores et al., 2012).

The 4 A test (4AT) (Tieges et al., 2021) is a rapid screening tool that requires no special training, making it suitable for initial screening in emergency departments, general wards, and nonintubated ICU patients. Its diagnostic specificity is slightly lower than that of the CAM-ICU (0.89 vs. 0.98), but its convenience makes it valuable for first-pass screening (Luetz et al., 2010). The Nursing Delirium Screening Scale (Nu-DESC) (Nydahl et al., 2022) and Delirium Detection Scale (DDS) (Otter et al., 2005) are also nursing-oriented tools, but they generally have lower validity and reliability than CAM-ICU does (van Eijk et al., 2009). The Mini-Mental State Examination (MMSE), developed for dementia assessment, has very low sensitivity (33%) for delirium diagnosis and is not recommended for delirium screening in critical care settings (van Eijk et al., 2009).

Notably, tools administered by family caregivers play a unique role in delirium assessment, particularly for patients with underlying cognitive impairment. The Informant Questionnaire on Cognitive Decline in the Elderly (IQCODE) collects information from informants familiar with the patient’s baseline cognitive status. When used in conjunction with the CAM-ICU, it aids in distinguishing three clinical states: (1) pure acute delirium (IQCODE negative, CAM positive); (2) delirium superimposed on dementia (IQCODE positive, CAM positive); and (3) chronic cognitive impairment without acute delirium (IQCODE positive, CAM negative) (Morandi et al., 2012). This differentiation holds clinical significance for nurses in developing personalized care strategies. An early study by Pisani et al., involving 130 ICU patients aged 65 years and older, revealed that screening for prior cognitive impairment in elderly ICU patients was effective (Pisani et al., 2003). Blandfort et al. reported that the abbreviated IQCODE had an area under the curve (AUC) of 0.72 for predicting delirium risk in hospitalized elderly patients, using a cutoff value of 3.3 (Blandfort et al., 2020). Burton et al. confirmed that the IQCODE has good sensitivity (0.75) for detecting long-term cognitive impairment following stroke, although its specificity is relatively low (Burton et al., 2021).

The family confusion assessment method (FAM-CAM) is another family-based tool validated for delirium detection. Mickle et al. reported that family members using the FAM-CAM detected 22.4% of POD cases, whereas 18.8% of cases were detected by research staff, indicating good applicability for caregiver-administered assessments (Mickle et al., 2025). The Chinese version of the FAM-CAM has also been validated in critically ill populations and is recommended as an auxiliary tool to support nurses in early delirium identification, especially during virtual visits (Xing et al., 2024). A systematic review by Zhou et al. reported a pooled sensitivity of 0.74 and specificity of 0.91 for FAM-CAM in delirium evaluation (Zhou et al., 2024), and it has also shown diagnostic value in emergency department patients with dementia (Mailhot et al., 2020).

In clinical practice, no single delirium assessment tool is suitable for all scenarios. Nurses should select the appropriate tool on the basis of patient characteristics (intubated vs. nonintubated, presence of baseline dementia), assessment purpose (routine screening vs. definitive diagnosis), and staff training level. For routine daily assessments of mechanically ventilated ICU patients, the CAM-ICU remains the most strongly evidence-based choice. For rapid initial screening or family-assisted assessment, the 4AT and FAM-CAM offer complementary value. Regular training and quality assurance are essential for maintaining assessment accuracy regardless of the tool used.

## 5 Nursing intervention methods

Nonpharmacological interventions are the cornerstone of delirium prevention and management in critically ill older adults, and nurses are the primary implementers of these strategies. Current clinical guidelines recommend multicomponent nonpharmacological interventions as first-line care for delirium, targeting modifiable risk factors through structured nursing practices. This section systematically reviews evidence-based nursing interventions organized around core care principles, including early risk identification, eCASH-oriented care, multicomponent in-hospital interventions, behavioral symptom management, and postdischarge transitional care (Tables 2, 3).

**Table 2:** Intervention measures and principles for cognitive impairment in elderly critically ill patients.

Category	Early identification	Early intervention
Main principles	Correction of factors affecting cognitive function	Hospital Elder Life Program (HELP)
	eCASH care	Multimodal analgesia: Behavioral Pain Scale (BPS), Critical-Care Pain Observation Tool (CPOT), Visual Analog Scale (VAS), Numerical Rating Scale (NRS) Maintenance of light sedation: Richmond Agitation-Sedation Scale (RASS)
	ICU interventions	Medication safety management (benzodiazepines, opioids, anticholinergics, systemic corticosteroids), early mobilization, sleep improvement, reduction of physical restraints, and reorientation training
Specific Measures of Multicomponent Nonpharmacological Interventions	Behavioral and psychological symptoms interventions	Music therapy, touch therapy, aromatherapy
	Home safety care	Fall prevention, management of dangerous items, and ensuring environmental familiarity

**Table 3:** Summary of key nursing interventions for delirium management in critically ill elderly patients.

Key interventions	Level of evidence	Principal findings	Clinical implications for nursing
Hospital Elder Life Program (HELP)	High	Meta-analyses demonstrate that HELP reduces delirium incidence by over 40% in hospitalized older adults and shortens hospital stay. The modified mHELP protocol reduces delirium probability by 56% in elderly surgical patients and generates long-term cost savings in care expenditure.	Nurses act as core implementers of HELP at the bedside. For ICU populations, priority should be given to modifiable risk factors (cognitive impairment, immobility, sleep deprivation, sensory deficits) with adaptive adjustments for illness severity.
eCASH care model (early comfort with analgesia, minimal sedation, humane care)	Moderate	Nurse-led eCASH sedation strategy reduces analgesic consumption, shortens mechanical ventilation duration, and optimizes pain control. Implementation of light sedation targets (RASS -1 to 0) is associated with reduced delirium duration and lower POD incidence in elderly postoperative patients.	Nurses undertake core responsibilities of systematic pain assessment (BPS/CPOT for unconscious patients, VAS/NRS for awake patients) and continuous RASS monitoring. Nurses communicate sedation efficacy to the multidisciplinary team to support physician titration of sedative regimens, without independent medication adjustment.
High-risk medication safety monitoring	High	Benzodiazepines, high-dose opioids, anticholinergic agents and systemic glucocorticoids are dose-dependently associated with elevated delirium risk in ICU patients.	Nurses conduct regular monitoring of consciousness and cognitive status, document cognitive changes associated with drug administration, promptly notify physicians of new-onset confusion, and advocate for medication regimen review when drug-induced delirium is suspected.
Early mobilization	High	Early physical and occupational therapy improves independent functional status at hospital discharge in mechanically ventilated patients. Multimodal exercise attenuates hospitalization-induced functional impairment and improves cognitive function in acutely ill very elderly patients.	Nurses should initiate mobilization as early as clinically feasible, graded from passive range-of-motion to assisted ambulation, with continuous hemodynamic monitoring. Exercise plans should be tailored to patient acuity and cognitive reserve.
Sleep optimization interventions	High	ICU patients with disrupted sleep architecture have significantly higher delirium risk. Randomized trials confirm that earplug plus eye mask interventions improve sleep quality and reduce delirium incidence. Circadian light regulation reduces sleep–wake cycle inversion.	Nurses should implement bundled sleep care: reduce nighttime noise and unnecessary awakenings, simulate natural light–dark cycles, provide earplugs/eye masks, and deliver relaxation interventions such as gentle back massage.
Physical restraint reduction	Moderate	Physical restraint use is an independent risk factor for delirium and posttraumatic stress disorder. Restraint reduction is associated with shorter hospital stays, with the most pronounced benefits in cognitively impaired patients.	Nurses should prioritize behavioral management over restraints. Alternatives include low-bed positions, bed alarm systems, increased family companionship, and purposeful activities. Restraints are only used as a last resort for immediate safety risks, with regular reassessment.
Reorientation training	Moderate	Environmental cue-based orientation activates hippocampal and prefrontal cortical function, supports cognitive recovery, and reduces disorientation in delirium-prone patients.	Nurses should provide consistent orientation information (date, location, caregiver identity), place clocks and calendars in the visual field, and use familiar personal items and family voice recordings to reinforce environmental familiarity.
Music therapy	Moderate	Individualized music listening reduces agitation severity and shortens delirium duration in elderly critically ill patients, via regulation of default mode network activity and circadian rhythms.	Nurses can implement individualized music interventions as an adjunctive strategy for agitation management, with volume and genre adjusted to patient preference and tolerance.
Gentle touch/hand massage therapy	Low	Gentle tactile interventions activate cutaneous mechanoreceptors and promote endorphin release, relieving anxiety and acute agitation in elderly acutely ill patients. Evidence is predominantly from small single-center studies.	Nurses may deliver standardized gentle hand massage as a low-cost, safe adjuvant measure for agitated patients, with attention to patient consent and cultural acceptability.
Aromatherapy	Low	Aromatherapeutic acupressure improves agitation symptoms in critically ill elderly patients. Large-scale high-quality RCTs in ICU populations remain limited.	Aromatherapy may be used as an optional complementary intervention. Nurses should assess for olfactory sensitivity and skin allergy risk before implementation.
Postdischarge home safety & transitional care	Low	Delirium recovery is gradual; patients with preexisting dementia rarely return to baseline cognitive function. Postdischarge disorientation elevates fall risk significantly.	Nurses provide structured discharge education on environmental modification, fall prevention and maintenance of familiar living environments. For patients with dementia, caregiver burden assessment and linkage to community care resources are needed.
Wearable monitoring devices	Low (preliminary)	Wearable sensors combined with machine learning show potential for continuous delirium risk monitoring and early detection of subtle changes. Most models are developed in general ward populations.	Wearable devices may assist nurses in continuous risk stratification in the future, but current evidence is insufficient for routine clinical use. Technical limitations (motion artifact, equipment interference) and workload impact require further evaluation.
Virtual reality (VR) assisted cognitive intervention	Low (preliminary)	Perioperative VR interventions show preliminary efficacy in reducing POD incidence and improving anxiety in surgical patients. ICU-specific evidence is extremely scarce.	VR may be considered as an adjunct for stable elderly ICU patients to promote early mobilization and cognitive stimulation. Infection control, patient tolerance and standardized ICU protocols remain to be established.
AI-assisted clinical decision support systems	Low (preliminary)	AI systems provide real-time delirium risk alerts and evidence-based intervention recommendations, with potential to improve guideline adherence. Most models are based on retrospective single-center data.	AI tools may support nurse-led delirium management in the future, but generalizability, EHR integration and data privacy barriers must be addressed before clinical translation.

Evidence levels were graded according to the GRADE (Grading of Recommendations Assessment, Development and Evaluation) framework, with consideration of study design, sample size, consistency of findings, and applicability to critically ill geriatric populations. High evidence: supported by multiple well-designed randomized controlled trials and high-quality systematic reviews/meta-analyses; moderate evidence: supported by limited randomized trials or consistent observational evidence; low evidence: predominantly small single-center studies or those extrapolated from non-ICU populations; very low evidence: preliminary exploratory evidence, insufficient for clinical recommendation.

### 5.1 Early risk identification and prevention strategies

Delayed identification of delirium is associated with poor clinical outcomes (Witlox et al., 2010). Given the age-related decline in brain reserve and the heterogeneity of physiological responses in elderly critically ill patients, early identification and targeted preventive interventions for high-risk individuals are core principles of delirium care (Jeyakanthan et al., 2026). The theory of brain plasticity suggests that early intervention can mitigate acute cerebral dysfunction and reduce long-term cognitive impairment, although this potential diminishes with advancing age (Mustafe Hidig and Nour, 2026).

The Hospital Elder Life Program (HELP) is a classic multicomponent prevention framework targeting key delirium risk factors: cognitive impairment, sleep deprivation, immobility, dehydration, and sensory deficits. Nurses play a central role in implementing HELP interventions at the bedside. A large randomized controlled trial demonstrated that the implementation of HELP in hospitalized older adults reduced the incidence of delirium by more than 40% (Inouye et al., 2006; Liang et al., 2021). A meta-analysis by Shen et al., which included 2,583 patients, revealed that compared with control treatment, HELP treatment significantly reduced the incidence of delirium and length of hospital stay (Shen et al., 2024). Additionally, a meta-analysis by Hshieh et al. revealed that HELP not only reduced the incidence of delirium but also saved $16,000 annually in long-term care costs (Hshieh et al., 2018).

To enhance clinical feasibility, a modified HELP (mHELP) protocol was developed, retaining core measures such as orientation support, nutritional support, and early mobilization. Kojaie-Bidgoli et al. validated through a double-blind randomized controlled trial that mHELP significantly reduces delirium risk and significantly shortens hospital stay (Kojaie-Bidgoli et al., 2021). A randomized clinical trial by Chen et al. also confirmed the efficacy of mHELP, with a 56% reduction in the probability of delirium among elderly patients who underwent abdominal surgery and who received mHELP (Chen et al., 2017).

Notably, the HELP was originally developed for general geriatric wards. In ICU settings, where patients have more severe illness and greater immobility, adaptive modifications are needed. Nurses should prioritize the most modifiable risk factors on the basis of individual patient circumstances to maximize preventive benefits.

### 5.2 Implementation of the eCASH care model

The eCASH model (Early Comfort using Analgesia, minimal sedation and maximal humane care) prioritizes patient-centered comfort through multimodal analgesia, light sedation, and humane care, with the aim of reducing the incidence of delirium among critically ill patients (Vincent et al., 2016). Nurses play a central role in delivering eCASH care, primarily through systematic assessment and continuous monitoring of pain and sedation status. A study conducted by Hui et al. demonstrated that among 149 mechanically ventilated patients in the ICU, the nurse-led eCASH sedation strategy significantly decreased analgesic consumption, shortened the duration of mechanical ventilation, and optimized pain control in mechanically ventilated patients compared with conventional physician-directed sedation (Hui et al., 2025).

Effective pain assessment serves as the foundation of eCASH care. For unconscious or mechanically ventilated patients, nurses adopt validated BPSs (including the BPS and Critical Care Pain Observation Tool [CPOT]) to evaluate pain responses during routine care and procedural interventions. For awake, communicative patients, the visual analog scale (VAS) and numeric rating scale (NRS) are applied for self-reported pain quantification (Devlin et al., 2018; Lewis et al., 2025; Gélinas, 2016). Nurses conduct periodic pain assessments in accordance with unit protocols and promptly notify attending physicians of inadequate pain control.

Maintaining light sedation constitutes another core nursing responsibility within the eCASH framework. The target level for light sedation corresponds to a Richmond Agitation–Sedation Scale (RASS) score of −1 to 0. At this level, patients remain awake, maintain eye contact, interact with nurses and family members, participate in mobilization therapy, and are able to rest undisturbed (Vincent et al., 2016; Hui et al., 2025). Nurses perform serial RASS assessments to identify signs of oversedation or agitation and communicate with the multidisciplinary team to adjust sedation regimens as needed.

Achieving and maintaining mild sedation and minimizing the risk of delirium depends not only on target-controlled monitoring, but also on the appropriate selection of sedatives and familiarity with the cognitive profiles of different drugs, enabling nurses to provide more targeted observational care (Jones et al., 2025). Dexmedetomidine, a highly selective α₂-adrenergic agonist (Weerink et al., 2017), produces cooperative and arousable sedation without significant respiratory depression, aligning inherently with the principles of eCASH, which emphasize maximizing patient interaction and humanized care (Vincent et al., 2016). Evidence from multiple randomized controlled trials and meta-analyses indicates that dexmedetomidine reduces the incidence and duration of delirium in mechanically ventilated ICU patients compared to benzodiazepines (e.g., midazolam) and propofol (Riker et al., 2009; Ruokonen et al., 2009; Heybati et al., 2022). However, in clinical practice, dexmedetomidine carries dose-dependent risks of bradycardia and transient hypotension due to its central sympathetic inhibitory effects (Zhang et al., 2026). These risks are more pronounced in elderly patients with underlying cardiovascular disease or during rapid loading dose infusions (Ng et al., 2019). Therefore, when administering dexmedetomidine, nurses should include continuous heart rate and blood pressure monitoring as core observational parameters and promptly report clinically significant bradycardia or hemodynamic instability to physicians. Propofol is the most commonly used intravenous sedative in clinical settings, characterized by rapid onset and recovery, and it has a lower delirium risk than benzodiazepines. However, it does not consistently demonstrate superior delirium protection compared to dexmedetomidine during prolonged high-dose infusions in vulnerable elderly populations (Lewis et al., 2022). In daily nursing practice, nurses should closely correlate sedative infusion rates with changes in RASS scores and cognitive status, and promptly report abnormal findings such as unexpected excessive sedation, paradoxical agitation, or withdrawal-related restlessness to the healthcare team to support individualized regimen adjustments.

Notably, the selection and titration of analgesic and sedative agents constitute medical decisions made by physicians. Nurses cannot independently prescribe or modify medication doses; instead, they provide vital bedside observational data to inform individualized therapeutic decisions. Clinical evidence confirms that implementation of the eCASH model is associated with a shortened duration of delirium and improved patient outcomes. For instance, a case report describing a patient receiving extracorporeal membrane oxygenation (ECMO) complicated by mixed delirium revealed that eCASH-guided nursing interventions effectively alleviated delirium symptoms (Acevedo-Nuevo et al., 2018). Studies focusing on elderly postoperative patients further indicate that nurse-led eCASH implementation reduces the incidence of POD (Shu-fang et al., 2017).

### 5.3 Specific measures for multicomponent nonpharmacological interventions (MNPI)

Multicomponent nonpharmacological interventions (MNPI) are currently recognized as the most effective nursing strategy for delirium prevention in critically ill patients. Because the pathophysiology of delirium involves multiple interacting pathways, single-target interventions often have limited efficacy in older critically ill patients with reduced physiological reserve. A network meta-analysis by Deng et al. confirmed that MNPI was superior to any single intervention in reducing delirium incidence and shortening ICU length of stay (Deng et al., 2020). Kasapoğlu et al. reported that nurse-led MNPI significantly reduced delirium severity and improved cognitive function in critically ill older adults (Kasapoğlu and Enç, 2022).

Nurses implement MNPI through five core approaches: medication safety monitoring, early mobilization, sleep optimization, physical restraint reduction, and reorientation training (Figure 1).

#### 5.3.1 Safety management of medications

Multiple classes of medications commonly used in the ICU, including benzodiazepines, opioid analgesics, anticholinergic agents, and systemic glucocorticoids, may increase the risk of delirium (Devlin et al., 2018). Nurses serve as frontline staff for medication safety monitoring and play a pivotal role in identifying medication-related cognitive changes.

Evidence indicates that benzodiazepines such as lorazepam and midazolam increase delirium risk in a dose-dependent manner and are also associated with a prolonged duration of mechanical ventilation and an extended ICU length of stay (Kok et al., 2018). Therefore, dexmedetomidine and propofol are commonly used as alternatives (Santos et al., 2025). Nevertheless, clinical nurses should be alerted that abrupt discontinuation after prolonged high-dose infusion may trigger withdrawal-related agitation and rebound delirium, which requires differentiation from primary delirium. In contrast, although propofol enables more rapid emergence, long-term high-dose administration carries risks of neurocognitive impairment in older adults, and its overall benefit for delirium prevention is inferior to that of dexmedetomidine. Although opioids are essential for analgesia, high doses may also prolong the duration of delirium (Gupta et al., 2020). Anticholinergic medications are well-recognized precipitants of delirium, and rivastigmine has been shown to increase rather than reduce mortality among patients with delirium (Burry et al., 2017; van Eijk et al., 2010). Systemic glucocorticoids can also significantly increase the risk of progression to delirium, possibly via neurotransmitter dysregulation (Schreiber et al., 2014).

Key nursing responsibilities in medication safety management include regular monitoring of consciousness level and cognitive status; documentation of changes in delirium symptoms associated with drug administration; timely notification of physicians regarding new-onset confusion or agitation; and advocating for review of medication regimens when drug-induced delirium is suspected. For patients with alcohol use disorder, nurses should closely monitor for withdrawal delirium and implement safety precautions during benzodiazepine tapering (Mainerova et al., 2015).

#### 5.3.2 Early mobilization

Early mobilization encompasses a graded spectrum of activities from passive range-of-motion exercises to assisted ambulation, tailored to patient acuity. Physical activity improves cerebral perfusion, activates neural plasticity, and reduces the risk of delirium and functional decline (Wang J. et al., 2020).

Schweickert et al. demonstrated in a landmark randomized trial that early physical and occupational therapy in mechanically ventilated patients significantly improved independent functional status at hospital discharge (Schweickert et al., 2009). Martínez-Velilla et al. reported that exercise intervention increased short physical performance battery scores by 2.2 points and Barthel index scores by 6.9 points in acutely hospitalized very elderly patients and attenuated hospitalization-induced functional impairment while improving cognitive function (Martínez-Velilla et al., 2019). Aerobic exercises such as walking and tai chi have also been shown to delay cognitive decline and reduce dementia risk among older adults (Voet et al., 2019).

Multicomponent exercise programs combining endurance, strength, and balance training provide more comprehensive benefits than single-mode exercise does, improving both cognitive outcomes and overall quality of life while reducing frailty (Casas-Herrero et al., 2019; Abdullah Alfadhel et al., 2020; Valenzuela et al., 2023). In ICU practice, nurses should initiate mobilization as early as clinically feasible, gradually increasing intensity under hemodynamic monitoring, and balancing safety with therapeutic benefit.

#### 5.3.3 Improving sleep conditions

ICU patients typically experience severe sleep architecture disruption, including markedly reduced rapid eye movement sleep and inverted sleep–wake cycles, with daytime sleep accounting for up to 50% of total sleep time. These abnormalities are driven primarily by environmental factors such as continuous noise, artificial lighting, and frequent nursing procedures, which disrupt circadian rhythm and increase the risk of delirium (Kang et al., 2023). Stallings et al. reported that abnormal sleep timing was associated with poor cognitive performance and a significantly high risk of delirium (Stollings et al., 2021).

The Society of Critical Care Medicine (SCCM) recommends multidimensional nonpharmacological sleep optimization as a core delirium prevention strategy (Devlin et al., 2018). Key nursing interventions include reducing nighttime noise and unnecessary awakenings; using earplugs and eye masks to create a sleep-conducive environment; simulating natural light–dark cycles with daytime bright light and nighttime dim lighting; and providing relaxation interventions such as gentle back massage (Mart et al., 2021; Obanor et al., 2021; Hsu et al., 2019). Randomized controlled trials have confirmed that earplug and eye mask interventions significantly improve sleep quality and reduce the incidence of delirium among ICU patients (Kiliç and Kav, 2023).

#### 5.3.4 Reducing physical restraints

Physical restraints such as bed rails and limb restraints are sometimes used to prevent unplanned extubation and self-injury in agitated patients. However, accumulating evidence has demonstrated that restraint use is an independent risk factor for delirium and posttraumatic stress disorder, which is likely due to restricted mobility, loss of autonomy, and psychological distress (Franks et al., 2021; Devlin et al., 2018).

A large-scale clinical study confirmed that reducing physical restraint use was associated with shorter hospital stays, with the most pronounced benefits in patients with cognitive impairment (Kwok et al., 2012). Nursing strategies to reduce restraint reliance include implementing continuous delirium assessment and behavioral symptom management; using low-bed positions and bed alarm systems as alternatives; increasing family visitation and companionship; and providing purposeful activities to reduce agitation (LaHue et al., 2021; Cui et al., 2022). Restraints should be used only as a last resort for immediate safety risk, with regular reassessment and prompt discontinuation when risk resolves (Adams et al., 2025).

#### 5.3.5 Reorientation training

Reorientation training aims to strengthen environmental cues and interactive information input to activate neural plasticity, helping patients maintain time, place, and personal orientation. It is among the most widely implemented delirium prevention nursing interventions (Munro et al., 2017). Nursing practices include providing consistent and clear orientation information such as date, location, and caregiver identity; displaying clocks and calendars in the patient’s visual field; using familiar personal items and family voices to reinforce environmental familiarity; and guiding patients to actively recall daily information through structured interaction. These interventions provide stable sensory input and activate hippocampal and prefrontal cortical function, supporting cognitive recovery (Munro et al., 2025; Johnson et al., 2024).

### 5.4 Management of behavioral and psychological symptoms

Acute behavioral and psychological symptoms such as irritability, agitation, sleep disturbances and perceptual abnormalities are common in patients with delirium, especially those complicated with underlying dementia. These symptoms increase the risks of unplanned extubation, falls and the use of physical restraints. Nonpharmacological nursing interventions include music therapy, tactile therapy and aromatherapy (Xu et al., 2025; Silva et al., 2018).

Music therapy may activate the default mode network of the brain, strengthening amygdala–hippocampal neural connections and regulating circadian rhythms to alleviate sleep disorders in patients with cognitive impairment (Mulia et al., 2025; Yuan et al., 2026). Recent intensive care-related studies have further verified that individualized music listening can reduce the severity of agitation and shorten the duration of delirium among elderly critically ill patients (Garcia Guerra et al., 2019; Dallıs et al., 2023).

Tactile therapy (such as gentle hand massage and therapeutic touch) may relieve anxiety and agitation by activating cutaneous mechanoreceptors and promoting endorphin release (Sert et al., 2026; Field, 2016). Updated systematic reviews confirm that gentle touch interventions delivered by nurses are a safe, low-cost adjunctive strategy for managing agitation in elderly patients with acute illness (Prandeh Afshar et al., 2025).

Aromatherapy alleviates acute behavioral symptoms by inhaling essential oils through the nasal cavity to modulate emotion-related brain regions and neurotransmitter levels; aromatherapeutic acupressure has been proven to improve agitation in critically ill elderly patients (Ebihara et al., 2021). At present, aromatherapy is being gradually adopted as an adjunctive intervention in intensive care settings, yet high-quality large-scale clinical trials targeting ICU populations are limited (Tan et al., 2023).

### 5.5 Home safety and transitional care

Cognitive recovery following delirium is a gradual process. For patients with preexisting dementia, complete restoration to the baseline cognitive level is rarely achieved even after the resolution of acute symptoms (Eshmawey, 2026). Following discharge, patients frequently continue to experience disorientation, impaired spatial navigation and difficulty recognizing their surroundings, placing them at high risk of falls. Nurses play a vital role in discharge education and the provision of transitional care guidance for family caregivers (Raghuraman et al., 2025).

Core home safety care guidelines include fall risk reduction via environmental modification: clearing clutter from floors; installing handrails in bathrooms and shower areas; rounding furniture corners or applying collision-proof strips; maintaining dry, nonslip flooring; and using antislip mats (Wang Y. et al., 2020; Livingston et al., 2020). Nurses should also advise caregivers to maintain a consistent living environment. Sudden changes to the home layout may trigger disorientation, increase fall risk and provoke agitation. Familiar personal belonging should be retained to support patients’ spatial orientation.

A more comprehensive transitional care plan should be developed for patients with underlying dementia, encompassing the assessment of caregiver burden, the coordination of follow-up care, and the linkage to community dementia care resources (Cumal et al., 2022).

## 6 Application of new technologies in nursing interventions for cognitive impairment

In recent years, digital health technologies have provided new tools for delirium prevention and management in critically ill older adults. Wearable devices, VR-assisted cognitive nursing, and AI-assisted clinical decision support systems have shown promising potential for improving assessment efficiency, enabling continuous monitoring, and enhancing intervention precision. However, most evidence remains at the preliminary research stage, and high-quality clinical validation in critical care settings, particularly from a nursing perspective, is still limited (Figure 1; Table 3).

### 6.1 Wearable devices

Wearable devices feature portability, continuous data collection and noninvasiveness. They can continuously capture physiological and behavioral data, including electrocardiogram, electromyography, actigraphy and electroencephalogram signals, and transmit such data to clinical management platforms in real time to generate dynamic trend graphs (Angelucci et al., 2025; Lv et al., 2025). In nursing practice, wearable devices enable uninterrupted monitoring of delirium risk, reduce the frequency of intermittent bedside assessments, and help nurses detect subtle early changes before overt symptoms emerge.

Available evidence supports the feasibility of wearable technology for delirium detection. Ahmed et al. reported that the combination of wearable sensors and machine learning algorithms could improve the accuracy of delirium diagnosis in patients with intracranial hemorrhage (Ahmed et al., 2023). Mao et al. developed a novel compact wearable electroencephalography device along with a matched automated analysis workflow to monitor continuous electroencephalography and predict delirium (Mao et al., 2026). Davoudi et al. adopted pervasive sensing technology and AI to collect data on patients and their surroundings using wearable sensors, light and sound sensors, and cameras, thereby realizing intelligent ICUs with autonomous monitoring functions (Davoudi et al., 2019). Actigraphy-based sleep monitoring can also objectively quantify sleep disturbances in ICU patients and assist nurses in adjusting sleep optimization strategies (Bate et al., 2023).

Despite the above advantages, several clinical limitations should be acknowledged. First, in ICU settings, the accuracy of wearable devices may be compromised by patient restlessness, electrode displacement and interference from other medical equipment. Second, most existing models are developed on the basis of general ward populations, and their performance among critically ill intubated patients requires further verification. Third, practical barriers, including equipment costs, staff training requirements, data security and increased nursing workload arising from data interpretation, still need to be addressed prior to widespread clinical application.

### 6.2 Cognitive nursing under VR technology

VR technology delivers immersive, multisensory interactive experiences that integrate visual, auditory and haptic feedback; activates the somatosensory cortex; strengthens cognitive neural networks; and improves attention and executive function (He et al., 2024).

In the field of delirium prevention, VR technology is applied mainly in two nursing scenarios: facilitating early mobilization and providing controlled cognitive stimulation to promote circadian rhythm regulation. Dong et al. implemented VR interventions for perioperative patients undergoing cardiac surgery (Dong et al., 2026). The results revealed that patients receiving VR interventions exhibited positive changes in anxiety, depression and sleep status over time, indicating that such interventions could reduce POD and improve selected psychological and functional outcomes in cardiac surgery patients. Luo et al. applied multiorgan VR technology to perioperative patients undergoing laparoscopic gastrointestinal surgery, which effectively reduced the incidence of POD within 3 days after surgery (Luo et al., 2026). Li et al. suggested that VR technology can effectively alleviate patients’ negative emotions, increase interest in and compliance with early mobilization, and positively affect cognitive functions such as working memory through immersive experiences, thereby lowering delirium risk across multiple dimensions (Li et al., 2025).

However, it should be noted that most existing research has been conducted in community or rehabilitation settings, and specialized evidence for ICU patient populations remains extremely limited. For stable critically ill elderly patients, VR-assisted sitting and standing activities may help reduce the risk of cognitive decline and delirium resulting from immobility; however, clinical efficacy data from randomized controlled trials in the ICU are still insufficient. The primary challenges facing the clinical application of VR technology include lower tolerance in hemodynamically unstable or severely agitated patients, reusable devices requiring compliance with infection control requirements, a lack of dedicated VR intervention protocols for ICU settings, and the need for specialized training for healthcare providers. Currently, VR should be regarded as an emerging adjunctive intervention with preliminary potential to be validated rather than a standard clinical care measure. High-quality large-scale clinical trials in intensive care settings are urgently needed to validate its efficacy and cost-effectiveness.

### 6.3 AI-assisted clinical decision-making systems

AI-assisted clinical decision support systems integrate multidimensional medical data using machine learning algorithms to increase the accuracy and timeliness of delirium risk prediction and management. For frontline nurses, these systems provide real-time delirium risk alerts, recommend evidence-based interventions, and improve guideline adherence, thereby reducing the variability in nursing quality caused by differences in clinical experience (Pandian et al., 2026).

An increasing body of evidence supports the clinical value of nurse-led AI-assisted delirium management programs. Zhang et al. reported a study protocol for a cluster-randomized controlled trial evaluating a nurse-led AI-assisted delirium prevention and management program in ICUs (Zhang et al., 2024). Further clinical trial results indicate that AI-anti-delirium interventions demonstrate favorable outcomes in terms of practicality, usability, attitudes, usage intention, and long-term effects (Zhang et al., 2025). Cho et al. implemented the ICU Delirium Automated Prediction (APREDEL-ICU) system, while it did not reduce the incidence of delirium, long-term use of the APREDEL-ICU is expected to increase the effectiveness of preventive care, leading to positive patient treatment outcomes (Cho et al., 2015).

However, several key limitations hinder their clinical translation. First, most current AI models are developed on the basis of retrospective single-center datasets, and their generalizability across diverse ICU populations with varying disease spectra remains uncertain. Second, seamless integration with existing electronic health record systems, data privacy protection, and regulatory approval represent practical barriers that need to be overcome. Future research should also focus on assessing the impact of AI systems on nursing workload and patient outcomes in real-world clinical settings.

### 6.4 Application of emerging technologies in daily nursing workflows

Despite the potential supportive value of wearable monitoring devices, virtual-reality-based cognitive training, and AI-assisted decision support, their successful translation into routine ICU nursing practice to deliver clinical value without increasing nursing workload requires seamless integration into existing nursing workflows, rather than being added as standalone tasks. Wearable devices should be used as continuous screening tools instead of replacing validated bedside scales; their data streams can be aggregated and summarized during hand-offs to support clinical judgement, with automatic artifact rejection to minimise false alarms. AI-generated delirium risk alerts should be filtered via clinical prioritisation algorithms to avoid exacerbating alert fatigue or increasing cognitive burden. For example, tiered notifications based on predictive probability thresholds should be integrated into existing electronic health record dashboards, so that nurses receive actionable, context-aware prompts instead of frequent low-value warnings. Virtual reality interventions are best administered to haemodynamically stable and cooperative patients during scheduled activities, under the supervision of dedicated nurses to guarantee safety and infection control. Critically, implementation must be accompanied by structured training, clear role definition, and iterative feedback loops from end-users. Future research should prioritize implementation science studies to evaluate the impacts of these technologies on nursing workload and patient outcomes in real-world ICU settings, ensuring digital tools can be effectively embedded into clinical practice.

## 7 Summary and future perspectives

This narrative review systematically integrates existing evidence on delirium care management for elderly critically ill patients, constructing a comprehensive care framework encompassing risk stratification, bedside assessment, in-hospital multicomponent interventions, behavioral symptom management, and postdischarge transitional care.

From a nursing perspective, early identification of modifiable risk factors is the first step in preventing delirium. Advanced age, social isolation, chronic comorbidities such as diabetes and stroke, surgery, high-risk medications, and systemic inflammation all increase the susceptibility to delirium in elderly critically ill patients. With respect to bedside assessment, the CAM-ICU remains an important tool for mechanically ventilated ICU patients, with its diagnostic accuracy validated. Family-informed tools such as the FAM-CAM and IQCODE offer unique complementary value by enabling nurses to distinguish between pure acute delirium and delirium superimposed on dementia, thereby supporting the development of individualized care plans.

Multicomponent nonpharmacological interventions include HELP-based preventive care, standardized pain and sedation assessment under the eCASH framework, medication safety monitoring, graded early mobilization, sleep environment optimization, a reduction in physical restraints, and orientation training. Adjunctive methods such as music therapy, gentle touch therapy, and aromatherapy are safe and low-cost measures for managing acute behavioral and psychological symptoms and are particularly beneficial for patients with underlying dementia. Postdischarge home safety guidance and transitional care are also integral components of the care continuum, aiding in fall risk reduction and maintenance of functional status, especially for patients with preexisting chronic cognitive impairment.

Emerging technologies such as wearable monitoring devices, VR-assisted cognitive interventions, and AI decision support systems demonstrate the potential to enhance assessment efficiency, enable continuous monitoring, and improve intervention precision. However, most evidence remains in the preliminary exploratory phase and lacks high-quality validation in real-world ICU care settings.

### 7.1 Limitations

This study has several limitations. First, as a narrative review rather than a systematic review incorporating quantitative meta-analysis, it inherently carries potential risks of publication bias in literature selection, and this review cannot provide combined quantitative estimates of intervention effectiveness. Second, the quality of supporting evidence varies across different intervention domains. Core strategies such as multicomponent interventions, HELP programs, and early mobilization are supported by high-quality randomized controlled trials and systematic reviews. In contrast, adjunctive interventions such as aromatherapy and touch therapy, as well as all emerging digital technologies, are primarily supported by small single-center studies. For certain interventions, high-quality evidence specifically targeting the ICU geriatric patient population remains limited, with many findings extrapolated from general geriatric medicine or rehabilitation settings. Third, most existing research focuses on short-term outcomes such as delirium incidence, duration, and hospital length of stay. Long-term follow-up data concerning cognitive prognosis, activities of daily living, caregiver burden, and cost-effectiveness are relatively scarce, hindering a comprehensive assessment of the long-term clinical and economic value of intervention strategies.

### 7.2 Future perspectives

Future research should prioritize the following directions to advance evidence-based delirium care for critically ill older patients. First, high-quality multicenter clinical trials with expanded sample sizes are needed. Future studies should extend follow-up periods to at least 1–3 years to evaluate long-term outcomes, including persistent cognitive impairment, functional status, readmission rates, and all-cause mortality. Concurrently, urgent subgroup analyses of patients with preexisting dementia are needed to develop tailored intervention protocols for this high-risk subgroup. Second, implementation science and health economics research should be strengthened. Future studies should explore barriers to and facilitators of the translation of evidence-based delirium care protocols into routine clinical practice, develop standardized nurse training programs, and assess the cost-effectiveness of intervention strategies from both healthcare system and societal perspectives. Third, further investigation into the clinical translation of digital health technologies is necessary. Future research should validate the diagnostic accuracy and clinical utility of wearable monitoring devices, VR-assisted interventions, and AI decision support systems within real-world ICU settings, with a particular focus on their integration with existing electronic health record workflows and impact on nursing workload. Additionally, specific ICU interventions and safety guidelines for older critically ill patients need to be established. Fourth, greater attention should be given to caregiver-centered transitional care. Future studies should develop structured nurse-led discharge education and home follow-up programs, evaluate their effectiveness in reducing postdischarge delirium recurrence and adverse events, and incorporate caregiver burden and quality of life as key outcome measures. Constructing a seamless hospital–community care continuum will be crucial for improving long-term prognosis in this vulnerable population.

## Glossary

4AT: 4 A’s Test
AI: artificial intelligence
BPS: Behavioral Pain Scale
CAM-ICU: Confusion Assessment Method for the Intensive Care Unit
CPOT: Critical-Care Pain Observation Tool
CPOT: C-reactive protein
DDS: Delirium Detection Score
DSM-5: Diagnostic and Statistical Manual of Mental Disorders, Fifth Edition
eCASH: early Comfort using Analgesia, minimal Sedatives and maximal Humane care
ECMO: extracorporeal membrane oxygenation
FAM-CAM: Family Confusion Assessment Method
GABA: gamma-aminobutyric acid
HELP: Hospital Elder Life Program
ICDSC: Intensive Care Delirium Screening Checklist
ICU: intensive care unit
IL-6: interleukin-6
IQCODE: Informant Questionnaire on Cognitive Decline in the Elderly
MCI: mild cognitive impairment
mHELP: modified Hospital Elder Life Program
MMP-9: matrix metalloproteinase-9
MMSE: Mini-Mental State Examination
MNPI: multicomponent nonpharmacological intervention
NLR: neutrophil-to-lymphocyte ratio
NRS: Numerical Rating Scale
Nu-DESC: Nursing Delirium Screening Scale
POD: postoperative delirium
RASS: Richmond Agitation-Sed
